# Lymphoma's manifestations in Sjögren's syndrome: is there a relation?

**DOI:** 10.1016/S1808-8694(15)31333-1

**Published:** 2015-10-20

**Authors:** Fabrícia Dias Colombano Limares, Rita De Cássia Soler, Ivo Bussoloti Filho

**Affiliations:** ^1^ Master studies in Otorhinolaryngology under course, Faculdade de Ciências Médicas de São Paulo, private practice; ^2^ Master and Ph.D., Faculdade de Ciências Médica da Santa Casa de São Paulo, Professor, Department of Otorhinolaryngology, Faculdade de Ciências Médica da Santa Casa de São Paulo; ^3^ Master and Ph.D. in Otorhinolaryngology. Escola Paulista de Medicina, Federal University of Sao Paulo, Joint Professor, Department of Otorhinolaryngology, Faculdade de Ciências Médica da Santa Casa de São Paulo

**Keywords:** Sjögren's syndrome, lymphoma, Sicca's syndrome

## Abstract

**S**jögren's Syndrome (SS) is considered a multisystemic chronic disorder, which is characterized by a lymphocytic infiltration of exocrine glands and autoantibodies production. **Aim**: Several studies have demonstrated increased incidence of Lymphoma in SS patients. Our study tries to determine this relation. **Study design**: Transversal cohort. **Material and Method**: Patients with Sicca's Syndrome from the Stomathology service, Santa Casa de Misericórdia Hospital, in Sao Paulo, ENT Department, from July 1999 to April 2002. **Results**: Out of 39 patients, 24 were diagnose as SS. Ages ranged from 19 to 83 years old, with predominance of women (69.7%). Time between the first symptoms and SS diagnosis were variable, ranging within 3.77 years. None of the analyzed patients developed lymphoma. **Conclusion**: Both SS early diagnosis and the increased risk of lymphoma's development in those patients are important, reason why long-term follow-up is essential. We observed that our findings were different from those in the literature. We did not detect any cases of lymphoma in our patients.

## INTRODUCTION

Sjögren's syndrome (SS) is considered a chronic, multisystemic affection that is characterized by exocrine gland dysfunction, systemic multiple organ abnormality and serological hyperreactivity. It presents two different forms: the primary form, characterized by xerostomia and xerophthalmia, and the secondary form in which at least one of these two symptoms is associated with connective tissue disease.

SS is characterized by lymphocytic infiltration in tissues and the production of autoantibodies. The etiology is not well defined but genetic, hormone and environmental factors may be involved^1^. Approximately 90% of the patients with SS are female subjects, aged between 40 and 50 years, but it has also been found in children^2^. The patients with enlarged salivary glands are constantly a great problem in SS. Persistent gland involvement in these subjects are of concern owing to the risk of transformation in Non-Hodgkin lymphoma. They are low-grade B cell lymphomas and they may have similar characteristics to the lymphomas that are developed in other mucosa-associated lymphoid tissues (MALT), which include the stomach, thyroid and liver^3^.

Malignant lymphoma was primarily described in patients with SS in 1963^4^. Subsequently, many described cases sustained the association between lymphoma and Sjögren's syndrome^5^,^6^ and recognized Non-Hodgkin Malignant Lymphoma (NHML) as the main complication of the disease progression^7^. The estimated risk of NHML was 44 times higher than in the normal population^7^.

According to Sreebney & Broid 1987^8^, patients with SS present higher risk of developing malignant lymphomas owing to the correlation between immune deficits and lymphoid system diseases.

The factors implied in the genesis of lymphoma include deregulation of mechanisms that lead to apoptosis, B cell hyperstimulation, and participation of infectious agents. Polyclonal lymphoproliferation that characterizes SS may be transformed into monoclonal and them into malignancy^9^. Among the considered infectious agents, there seems to be a correlation between hepatitis C virus and SS^10^.

The concept of MALT lymphoma suggests that these tumors correspond to stages of post-follicular cell differentiation and normally they are present in sites of chronic antigen stimulation firing by persistent infections or autoimmune processes or both^11^.

Salivary MALT lymphomas may be of low grade and indolent^12^,^13^. Some patients survive years without any evidence of systemic disease after the diagnosis and many salivary lymphoepithelial lesions that have evidence of monoclonality do not progress to definite manifestations of lymphoma^13^. Studies have shown that between 40 to 80% of the salivary lymphoepithelial lesions may have monoclonal proliferation^14^.

Five percent of the patients with primary SS occasionally develop B cell lymphoma^15^.

According to Zufferey et al.^16^, the interval between diagnosis of SS and onset of lymphoma normally ranges from 4 to 12 years.

## MATERIAL AND METHOD

We carried out a prospective clinical and laboratory cross-sectional study with 39 patients complaining of xerostomia in the period between July 1999 and April 2002, in the ambulatory of Stomathology, Department of Otorhinolaryngology, Santa Casa de Misericórdia de Sao Paulo. As inclusion criteria, all patients presented initial complaint of dry mouth (xerostomia). There was no exclusion of patients that were under use of any type of medication, nor patients that had associated diseases.

Patients were classified as having Sjögren's syndrome based on GECE criteria17., 18., 19., 20.. The criteria included 6 items out of which 4 should be present to define the classification.

## RESULTS

A sequential sampling was made with 39 patients, all with xerostomia, and out of the total, 24 were classified as having SS (they complied with 4 out of 6 criteria) compared to 15 patients that were not classified as having Sjögren's syndrome (NSS) (Tables 1 and 2). The age of the patients ranged from 19 to 83 years. We observed that among the 15 patients that did not have SS the mean age was 53.67 years and among the 24 holders of SS the mean age was 61.29 years. The time interval between beginning of xerostomia and diagnosis of SS ranged from 6 months to 13 years, mean time of 3.77 years.

As to gender, in our sample we found predominance of female patients in relation to male patients. Out of the total number of male patients only 16.7% had SS, whereas 69.7% of female patients had SS.

In our sample, we did not observe development of lymphoma in any of the assessed patients.

## DISCUSSION

Low prevalence of primary Sjögren's syndrome in the general population, estimated to be 0.2 to 1.4%^21^, combined with the estimated risk of MALT lymphoma, reaching 6.4 cases per 1,000 per year^7^ emphasizes the limited area of study of the disease owing to reduced number of patients^22^.

Lymphomas affect many different extranodal sites, normally in the gastrointestinal tract, appearing in sites of chronic inflammatory disease or autoimmune diseases, such as Hashimoto thyroiditis, Sjögren's syndrome, Helicobacter Pylori inducing chronic gastritis, etc.^23^. The lesion tends to remain stable for prolonged periods of time without dissemination, and it also tends to involve other mucous organs^24^.

A recent report informed that MALT lymphoma amounts to 46 to 56% of malignant lymphomas that were developed in patients with SS23., 25..

The occurrence of MALT lymphoma in the gastrointestinal tract is less common in patients that have preexisting SS^26^.

According to Tomani et al. (2002)^26^, 5 patients presented MALT lymphoma at the same time in which primary SS was diagnosed or they developed lymphoma after the diagnosis of SS. In 3 patients, Sjögren's syndrome was diagnosed 1, 3 and 10 years, respectively, before the diagnosis of MALT lymphoma. In the other 2 cases, SS and MALT lymphoma were diagnosed simultaneously. Consequently, for these 5 patients, the time interval between diagnosis of SS and diagnosis of MALT lymphoma was 2.8 years.

Royer et al. (1997)^27^ reported 9 patients with MALT lymphoma associated with SS and in 5 the parotid gland was the most frequent initial site, followed by stomach, lung, oral mucosa and lymphoid nodes.

Sutcliffe et al., (1998)^12^, in a retrospective study with 72 patients with primary SS that were classified according to the European criteria of Vitalli et al., followed up for a period of 10 years, had 5 patients that developed MALT Non-Hodgkin lymphoma (prevalence of 7%) and they were all women. As to lymphoma diagnosis, the age was 63 years and the mean disease time was 14 years. According to the study, the likelihood of MALT lymphoma development was 18% after 20 years from onset of the disease.

Sutcliffe et al. (1998)^12^ observed the presence of extraglandular manifestations as opposed to Sicca Syndrome alone, which does not differentiate the lymphoma group, because both groups have high frequency of extraglandular involvement.

In their study, Voulgarelis et al. (1999)^22^ suggested that salivary glands in patients with SS are the most commonly affected initial sites in the transformation of neoplastic B cells, but other MALT sites, such as bone marrow and lymphoid nodes were also identified as initial sites of malignant transformation.

Recent studied identified predictive factors for the occurrence of Non-Hodgkin lymphoma in patients with SS: parothydomegalia7., 27., low doses of parotid radiation^27^, splenomegalia27., 28., lymphadenopathy7., 27., 28., presence of urinary or serum monoclonal component27., 28., reduction of polyclonal serum immunoglobulin27., 28., reduction of rheumatoid factor titers^28^, leukopenia^28^ and purpura^28^. In our study, we did not observe any of the above mentioned manifestations.

According to Voulgarelis et al. (2001)^28^, the presence of mixed monoclonal cryoglobulin proves to be the most significant risk for development of lymphoma.

In our study, what attracted our attention was the fact that even though all SS patients had been followed up to present, we have not found any clinical or laboratory indication of lymphoma development, contrarily to the literature, which makes us pose the following question: would the pathology behave differently in Brazil?

## CONCLUSION

As years go by, we have observed an increasingly higher number of patients with Sjögren's syndrome owing to the redefinition of classification criteria and the new techniques that favor more precise diagnoses. We emphasize the importance of SS diagnosis and the risk of malignant development with time, which means that long-term follow-up is absolutely mandatory in such patients. In our study, patients have been followed up to present and there have been no clinical or laboratory manifestations for the diagnosis of lymphoma.
